# Cell Therapy: A New Section for the Transplant and Cell Therapy Community

**DOI:** 10.3390/curroncol28060402

**Published:** 2021-11-16

**Authors:** David Allan

**Affiliations:** Department of Medicine, The Ottawa Hospital and University of Ottawa, Ottawa, ON K1N 6N5, Canada; daallan@toh.ca

The new Cell Therapy section [1] in *Current Oncology* will strive to report timely and important studies in the rapidly evolving field of cell therapy to treat cancer. As the new Section Editor-in-Chief, I want to welcome you to this section on behalf of our newly established editorial board and associate section editors. The launch of this section is timely and will strive to publish landmark articles that will map our progress towards improved cell-based treatments in cancer. We can anticipate more refined cellular therapies with enhanced efficacy and reduced toxicity for an expanded list of indications in the near future. Moreover, innovations and developments in conventional hematopoietic cell therapy continue to allow access to this established life-saving treatment for new patient groups, with less morbidity and mortality and with greater therapeutic value. Efforts continue to ensure greater access to better donors, improved prevention and management of complications, and refinements in patient selection and disease monitoring that will improve outcomes for future patients. New approaches in immune effector cell therapy, including chimeric antigen-receptor-based products, will enhance their efficacy, reduce their toxicity, and we can expect an expansion to new indications across the spectrum of hematologic malignancies and solid tumors. A venue for sharing and collating studies and topics on cellular therapy for cancer will allow greater exposure and sharing of new evidence, and greater knowledge translation so discoveries can be developed into accessible treatments for more patients sooner. 

There are key issues that will need special attention as we move forward in the field of cell therapy and we hope to usher in the next era of studies that will accelerate further progress towards more widespread availability to patients in need. Continued attention to robust product characterization remains paramount for several reasons. Reproducibility and progress towards regulatory approval of new products or new indications for treatment will continue to rely heavily on careful and detailed product characterization. Could more centralized or standardized collection and processing methods be increasingly important to limit the regulatory burden and enhance the quality of products in the future? As many cell-based studies are modest in size due to the resource-intensive nature of producing cellular products and with the complexity of launching studies and accruing study subjects, the emergence of evidence that supports safety and efficacy may increasingly rely on pooling of results through meta-analysis which will be most meaningful when outcome reporting is similar between studies. Master protocols for specific types of cell-therapy approaches may even be needed to accelerate the generation of knowledge sooner and make the transition to larger studies that meet regulatory approval earlier. Minimizing the risk of bias in publications is another work in progress and increasing attention with the emergence of reporting guidelines and tools to assess for potential bias should allow initial observations to be translated more often and more predictably to larger scale trials. 

Our collective new space for sharing knowledge in Cell Therapy in *Current Oncology* will expand our capacity for knowledge-sharing and stimulate the reporting of new innovations that will propel this field forward. Studies that address preclinical animal studies that strive to bridge the gap from discovery to patient care will be welcome, early phase trials that are proof-of-principle, along with retrospective and registry-based studies will also be included in this new section of the journal as part of the continuum of clinical research. Prospective studies and randomized controlled trials along with knowledge synthesis work that involves meta-analyses will also be encouraged and will push our field towards more widespread clinical use. Our editorial team is looking forward to using this space within *Current Oncology* to present studies and topics on cell therapy in the treatment of cancer. We are pleased with our partnership and close alliance with Cell Therapy & Transplant Canada and also welcome researchers and collaborators from the international community to share their work through our new section on Cell Therapy.
